# Machine learning based approach to exam cheating detection

**DOI:** 10.1371/journal.pone.0254340

**Published:** 2021-08-04

**Authors:** Firuz Kamalov, Hana Sulieman, David Santandreu Calonge

**Affiliations:** 1 Department of Electrical Engineering, Canadian University Dubai, Dubai, UAE; 2 Department of Mathematics and Statistics, American University of Sharjah, Sharjah, UAE; 3 Department of Communication and Media, Canadian University Dubai, Dubai, UAE; KTH Royal Institute of Technology, SWEDEN

## Abstract

The COVID-19 pandemic has impelled the majority of schools and universities around the world to switch to remote teaching. One of the greatest challenges in online education is preserving the academic integrity of student assessments. The lack of direct supervision by instructors during final examinations poses a significant risk of academic misconduct. In this paper, we propose a new approach to detecting potential cases of cheating on the final exam using machine learning techniques. We treat the issue of identifying the potential cases of cheating as an outlier detection problem. We use students’ continuous assessment results to identify abnormal scores on the final exam. However, unlike a standard outlier detection task in machine learning, the student assessment data requires us to consider its sequential nature. We address this issue by applying recurrent neural networks together with anomaly detection algorithms. Numerical experiments on a range of datasets show that the proposed method achieves a remarkably high level of accuracy in detecting cases of cheating on the exam. We believe that the proposed method would be an effective tool for academics and administrators interested in preserving the academic integrity of course assessments.

## 1 Introduction

The COVID-19 pandemic has brought unprecedented challenges to the way schools and universities facilitate learning. Due to imposed quarantine measures, educational institutions around the world were left with no alternative other than to continue their course delivery in an online format. Although remote teaching has been adopted by many tertiary institutions, it suffers from several significant drawbacks. One of the main challenges is ensuring academic integrity. Students who take their exams at home are expected to work independently without use of any external help. However, in practice, a nontrivial portion of students attempt to circumvent the rules of academic integrity by, for instance, using digital cheating or contract cheating [1, 2] i.e., remunerating a third-party to do the work on their behalf. Universities try to combat exam cheating with the use of remote proctoring, webcams, LockDown Browser (Respondus), plagiarism software (e.g., Turnitin, SafeAssign, iThenticate, to cite a few) and monitoring software during the exam. Nevertheless, it remains relatively effortless for a student to receive third-party aid during the exam, as indicated by a University of New South Wales report, which found that 139 science students had “hired ghost writers from the Chinese messaging site WeChat to complete their work” [3]. Our goal in this paper is to identify cases of cheating on exams based on a post-exam analysis of the scores to detect abnormal scores. The significance of our approach lies in the novel use of machine learning techniques to identify the anomalous grades on the exam.

Studies over the past two decades in various countries have provided important information on the prevalence of academic dishonesty in higher education [4–8]. Results of a study by Lanier [7] of 1,262 students taking courses in regular and distance learning formats indicated for instance that cheating was much more widespread in the online sessions. Chirumamilla et al. [5], in a survey of 212 students and 162 teachers in Norway, identified six most commonly used cheating practices: impersonation, forbidden aids, peeking, peer collaboration, outside assistance and student–staff collusion. In the Canadian context, Eaton [9] investigated cases of academic integrity violations in the higher education sector, covered by the media between 2010 and 2019. The report concluded that academic misconduct was a “large issue” and that deeper investigation into its extent was warranted.

There is a growing body of literature that recognizes post-exam grade analysis as one promising avenue for detecting exam violations [10, 11]. By comparing students’ continuous assessment grades to the grades on the final exam, exam violations can be identified. Significant deviations from the expected results may be an indication of potential exam transgression. Sudden and unexpected high scores on the final exam for an average student may raise a few eyebrows, red flags and be considered abnormal. However, the situation is not always as straightforward as it seems. If the final exam is relatively unchallenging and the majority of students receive high grades, any violation would be less observable. In addition, it is important to take into account the sequential nature of course assessments and that the order of the scores matters. The temporal order consideration complicates the situation considerably. One popular algorithm to analyze ordered data is a recurrent neural network [12]. It allows for information to propagate through a sequence via parameter sharing.

In this study, we attempt to identify anomalous scores on the final exam using a combination of a recurrent neural network and an outlier detection method. The proposed method uses data consisting of student course assessments including quizzes, the midterm exam, and the final exam. Outlier detection methods are effective in identifying points in data that deviate from the general population and are often used in fraud detection. Recurrent neural networks are used to process sequential data and can be applied to chronologically ordered data such as course assessments. First, the data is processed by a recurrent neural network. Then, the output of the neural network is fed into an outlier detection method which determines the potential instances of cheating. The resulting algorithm provides a robust and efficient tool to identify potential cases of academic violation. We are optimistic that the proposed method would be a useful tool in the fight against exam fraud.

This study is crucial as educational institutions around the globe are looking into effective ways of preventing academic misconduct with the use of advanced analytics such as artificial intelligence. According to Senthil Nathan, managing director and co-founder of Edu Alliance Ltd, “Distance education has not been widely accredited in this region, mainly because of authentication issues” [13]. Recent developments in the field of remote learning have led to a renewed interest in detecting academic dishonesty in online environments, with the use of behavioral biometrics, learning analytics, data forensics [14] or data mining [15]. A search of the literature however revealed few studies (apart perhaps from [16]) which addressed this issue using anomaly detection methods. The experimental work presented here provides an alternative approach for identifying potential cases of exam dishonesty based on student scores using machine learning methods.

Despite the effectiveness of the proposed algorithm, the generalizability of these results is subject to certain limitations. It is certainly plausible and possible for a student to achieve an unusually high score through hard work and study. Therefore, any case identified as a potential violation requires further investigation by a human expert before a final decision is declared. Notwithstanding the limitations, this work offers valuable insights into final exam fraud detection and aims to contribute to this growing area of research.

Our paper is organized as follows. Section 2 provides an overview of existing literature on fraud detection and related topics. In Section 3, we present our methodology. We describe the details of the proposed approach to identify potential cases of exam violations. In Section 4, we carry out a range of numerical experiments to test the performance of our method. We conclude the paper with a closing summary and remarks in Section 5.

## 2 Literature

“Are Online Exams an Invitation to Cheat?” [17]. Academic dishonesty in Higher Education during online final exams is prevalent and far from being a new occurrence. A decade ago, Carnevale [18] had already argued that technology was “offering students new and easier ways to cheat” (para.1). The findings of a study by King, Guyette Jr., and Piotrowski [19] on the attitude and behavior of business students towards cheating in an exam administered online indicated that 73.6% of the respondents perceived that cheating online was an easy task (p.7). Since the switch to online examinations, as a consequence of the pandemic lockdown, there seems to be an unwelcome recrudescence of student academic misconduct cases [20]. In the U.S., Boston University reported for instance that students had “… used various means, including websites such as Chegg, to get help during the quizzes given remotely” [21].

Outlier detection is a well investigated aspect of data science [22]. Anomaly detection has been used successfully in many applications. For example, medical claims processing involves large volumes of data which necessitates the use of automated screening procedures. Thus, anomaly detection algorithms are well suited to identify potential fraudulent claims [23, 24]. Similarly, financial data processing such as credit card transactions requires automatic means of anomaly detection [25]. In network security, anomaly detection is used to identify malicious signals in the network traffic [26]. Recently, investigators in education have examined outlier detection methods to efficiently identify students’ irregular learning processes [27, 28], typing patterns [29], or cheating in Massive Open Online Courses [30].

Outlier detection methods can be divided into two groups: semi-supervised and unsupervised methods. In a semi-supervised outlier detection method, an initial dataset representing the population of negative (non-outlier) observations is available. A machine learning tool such as one-class SVM can be trained to obtain the boundary of the distribution of the initial observations. Then new observations are categorized according to their distance from the boundary. In unsupervised methods, the algorithm is trained without a clean initial dataset of negative observations. The majority of the algorithms fall under the unsupervised category. Unsupervised anomaly detection methods can be grouped into model, distance, and density-based approaches. An up-to-date review and analysis of the modern methods can be found in [31]. Model-based approaches are the simplest methods for outlier detection. They are based on the assumption that the normal data is generated according to some statistical distribution [32, 33]. The distribution parameters such as the mean and standard deviation are calculated based on the sample data. More sophisticated approaches use kernel functions to estimate the underlying distribution of data [34]. Then the points with low probability are deemed to be outliers. Distance-based approaches operate on the assumption that abnormal points are far from the main cluster of points. A popular distance method uses a heuristically-determined radius *δ* and percentage *p*. Then a point *x* is considered an outlier if at most *p* percent of all other points have a distance to *x* less than *δ* [35]. Density-based approaches compare the data density at the given point relative to the density at the neighboring points. Points with relatively low density are considered anomalous. One of the popular density methods is the local outlier factor (LOF) which computes the local density at a given point. The points with a lower local density compared to their neighbors are considered outliers [36]. A variant of the LOF was used in [37] to identify schools with unusual performance on standardized tests.

Since the number of anomalous scores is relatively low, it leads to the issue of imbalanced class distribution. Traditional machine learning techniques are not well-equipped to handle imbalanced data [38]. There exists a number of ways to deal with imbalanced data. A popular method called SMOTE achieves data balance by artificially adding new minority points to the original dataset prior to the training phase [39]. More recently a new method based on gamma distribution was proposed to generate new instances of minority points [40].

## 3 Methodology

In this section, we present our algorithm for detecting potential cases of cheating on the final exam. The inputs to the algorithm are sequences of grades—quizzes, midterm exam, the final exam—of an entire class, while the output is a collection of labels—one label per student—indicating whether each student cheated or not. The proposed method consists of two parts: regression and unsupervised outlier detection. First, a recurrent neural network model is trained to predict the final exam scores based on the previous assessment scores. Then an outlier detection model is applied to identify the instances where the difference between the actual and the predicted final exam scores is abnormal. Since the input data is unlabeled, the proposed method is an unsupervised algorithm.

**Fig 1:**
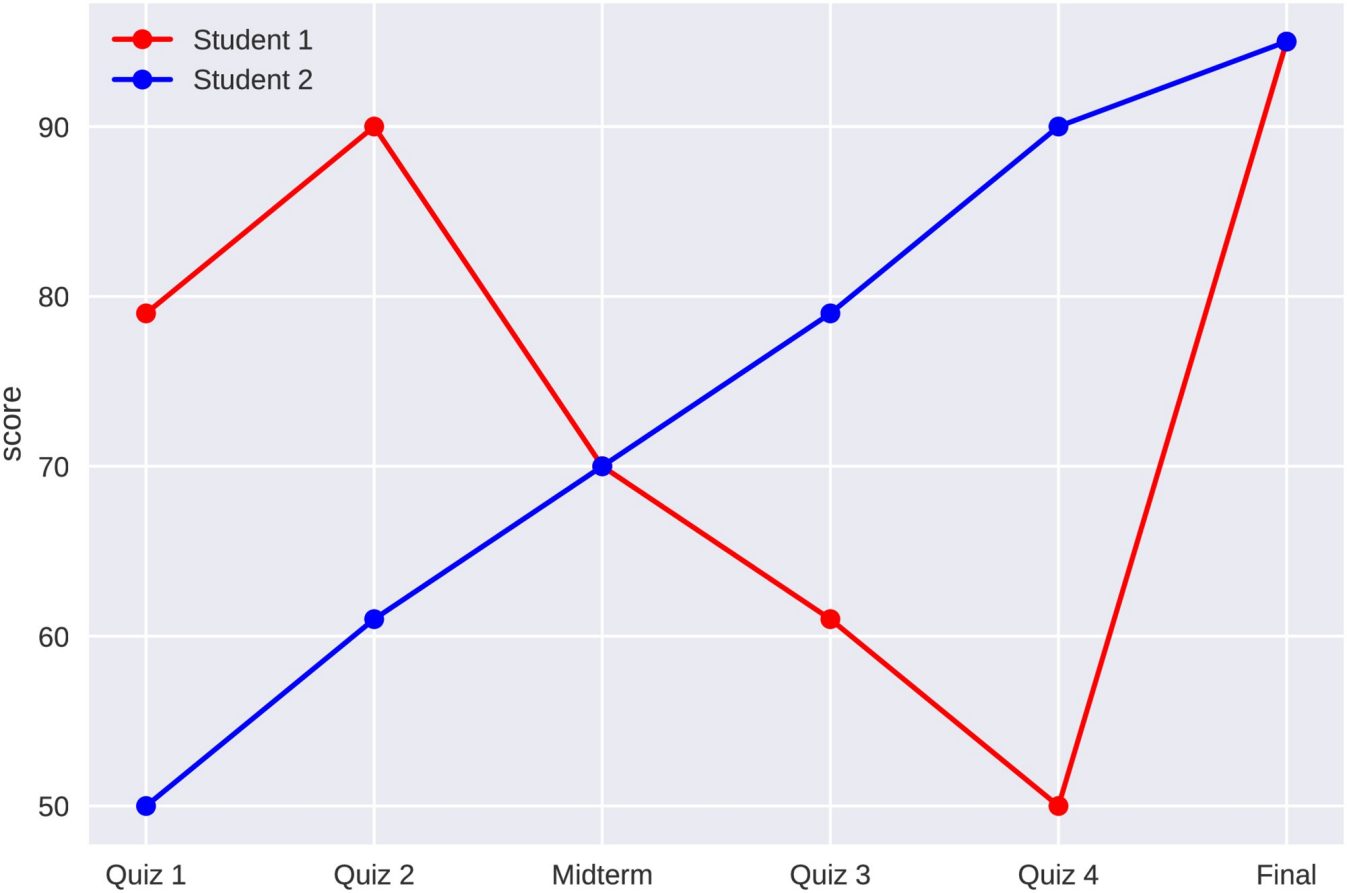
Both sequences of scores consist of the same values, but in different order. The steady progression of grades of Student 2 makes a score of 95 on the final exam seem plausible. On the other hand, the pattern of grades for Student 1 makes a grade of 95 on the final exam highly unexpected.

The job of identifying abnormal final exam scores is not a straightforward task. There are several considerations that need to be taken into account. The anomaly of a final exam score depends uniquely on all the other scores in class. An exam score that is anomalous for one class of students may be completely normal for another class. However, further experiments are required to better understand this issue. Second, the existing machine learning techniques must be tailored to the particular features of the problem. We wish to identify the anomalous scores by comparing the scores prior to the final exam to the score on the final exam. It stands to reason that a student with a large gap between the two scores is more likely to have cheated. Implementing this philosophy requires a custom solution. Third, the temporal nature of the assessments must be taken into account during the analysis. Quizzes, term exams, projects, and the final exam are taken in sequence. The order of the assessments contains crucial information. For example, the scores {79, 90, 70, 61, 50, 95} convey different information than the scores {50, 61, 70, 79, 90, 95}. The former sequence of scores is normal whereas the latter is abnormal (Fig 1).

Since the scores in different classes have their own particular characteristics the grades of each class must be analyzed individually. The need for individual analysis of each set of exam scores steers us in the direction of outlier detection methods. Traditional outlier detection algorithms are designed to function without any prior training. Each dataset is analyzed on its own and the abnormal instances are flagged by the algorithm. However, the standard outlier detection algorithms suffer from two crucial drawbacks. First, given an *n*-dimensional feature vector, unsupervised outlier detection methods do not always identify the important features. It is possible that one of the features is more relevant than the rest in identifying outliers. In our case, it is clear that the grade on the final exam is more relevant than, say, the grade on quiz 1. The traditional outlier detection methods do not provide an option of assigning different weights to features. This disadvantage applies to many machine learning algorithms including neural networks. Second, outlier detection methods do not take into account the temporal nature of the sequential data. The temporal information can significantly improve the learning outcomes of algorithms. Given the above deficiencies, outlier detection methods must be complemented with another method that takes into account the outlined considerations. One of the most effective modern machine learning approaches to handling sequential data is recurrent neural networks. These networks use the data from the previous steps to make predictions about the next step. Thus, our approach is based on two key ingredients: neural networks and anomaly detection.

### 3.1 Anomaly detection

**Fig 2:**
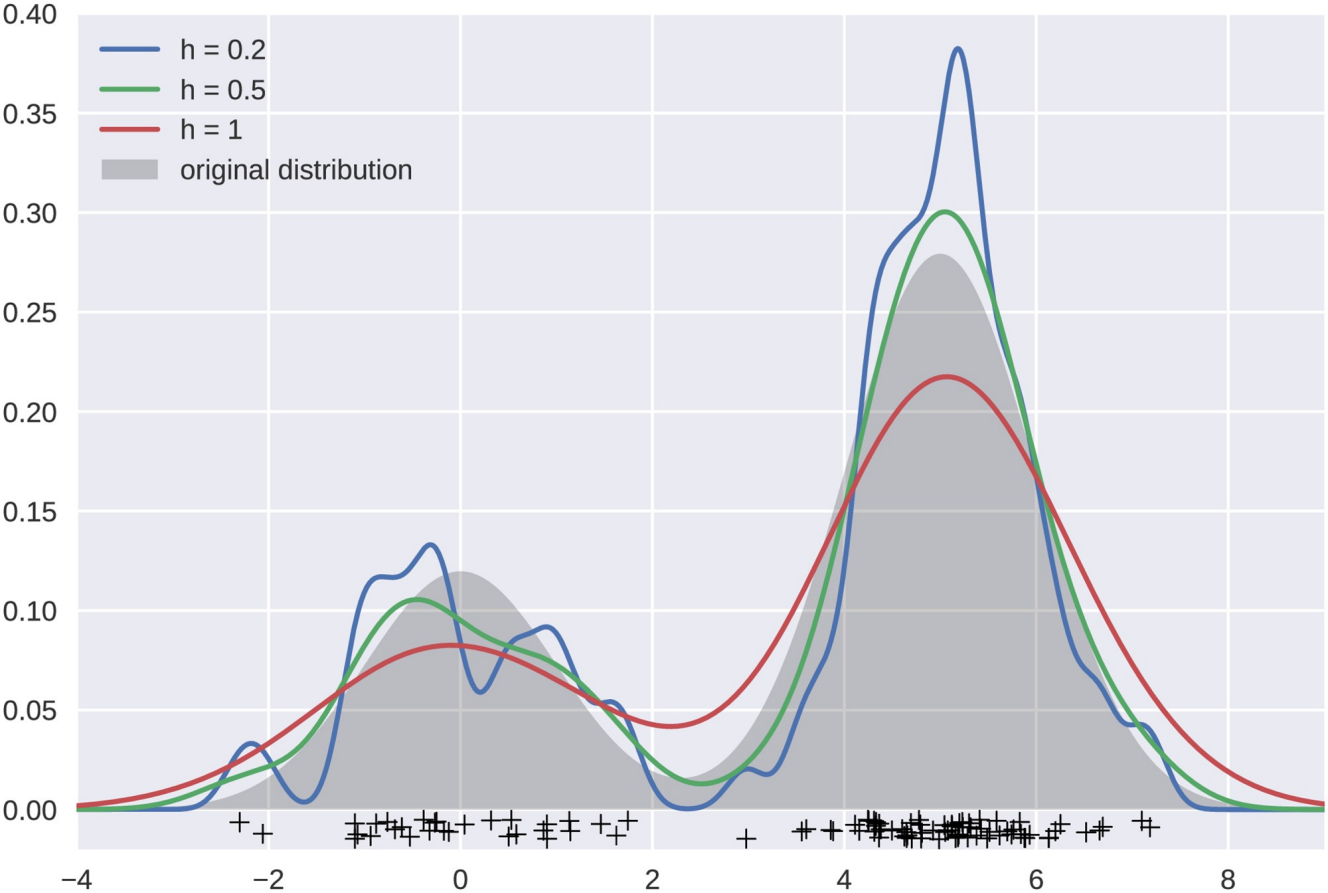
1D kernel density estimate of a Gaussian distribution using various bandwidth values.

As mentioned earlier in Section 1, anomaly (outlier) detection algorithms are widely used in many applications including fraud detection, medical diagnostics, network security, and others. In this paper, we employ a kernel density-based method to identify the outliers in a dataset. Kernel density estimation (KDE) is a well-established statistical method that is widely used in different applications [41]. It is a nonparametric technique for estimating the underlying distribution of the sample data using a kernel function. Concretely, the distribution of the data is approximated by the average of the kernel functions taken over the entire dataset. Let {*x*_1_, *x*_2_, …, *x*_*n*_} be an i.i.d. sample drawn from an unknown probability density function *f*. Then the kernel density estimate of *f* is given by

$$\begin{array}{c} \tilde{f}\left(x\right)=\frac{1}{n}\sum_{i=1}^{n}K_{h}\left(x-x_{i}\right), \end{array}$$ (1)

where *K* is the kernel function, *h* is the bandwidth parameter, and $K_{h}\left(t\right)=\frac{1}{h}K\left(\frac{t}{h}\right)$. There exists a number of kernel functions. The most popular kernel is the Gaussian function i.e.

$$\begin{array}{c} K(t)=\phi (t), \end{array}$$ (2)

where *ϕ* is the standard normal density distribution. As shown in Fig 2, the bandwidth parameter *h* controls the smoothness of the density function estimate as well as the trade-off between the bias and variance. Upon estimating the underlying distribution of the dataset, we can use it to calculate the probability of a given data point being a normal point. The points with low probability are deemed as outliers.

### 3.2 Long short-term memory

Recurrent neural networks (RNNs) and their subclass long short-term memory networks (LSTMs) are a natural choice to deal with sequential data. RNNs and LSTMs are designed to deal efficiently with sequence data by allowing previous outputs to be used as inputs. They have been used successfully in various applications including speech and text recognition as well as time series analysis such as daily stock price data [42]. We use LSTMs in the proposed algorithm. We refer the reader to [43] for the details of the LSTM architecture.

### 3.3 Algorithm

Our goal is to design an algorithm that accomplishes the following two fundamental tasks:

1. Identify students whose final exam grade is unreasonably high relative to the rest of the class and their prior assessment scores.
2. Take into account the sequential nature of the data under consideration.

We employ LSTMs to accomplish the second task. Concretely, we build a regression model using an LSTM network with the aim of predicting the final exam score based on the scores prior to the final exam. The input variables in our model include scores on quizzes, midterm exam, projects, and other pre-final exam assessments. The output of the model is the score on the final exam. The model is trained to minimize the mean squared error of the predictions. The neural network architecture for predicting the final exam scores consists of an input, output, and hidden layers. The size of the input layer depends on the dataset. In our numerical experiments, the input layer has size 5 which corresponds to four quizzes and one midterm exam scores. The output layer has size one which corresponds to the final exam score. The student scores are processed by the three hidden layers to establish the weights of the network to minimize the prediction error. The first hidden layer is a LSTM layer and the other two layers are fully-connected layers. The neural network was constructed using Keras [44]. The details of the network layers are presented in Table 1.

**Table 1:** The neural network architecture for the proposed algorithm.

	Hidden layer 1	Hidden layer 2	Hidden layer 3
Type	LSTM	Fully connected	Fully connected
Dimension	8	64	32

The exam scores predicted by the LSTM regression model are compared to the actual exam scores. Next, we apply a KDE-based outlier detection method described in the preceding section to identify the potential cheating cases on the basis of the predicted and actual exam scores. We apply Scott’s rule to determine the bandwidth parameter for the KDE. To identify the outliers, we select 9% of the instances with the lowest probability based on the estimated density function. The contamination level was determined based on the fact that 10/110 of the instances in the data are outliers. In general, the contamination level is set by the expert based on the expected number of outliers within the data. The synopsis of the proposed detection procedure is given below.

**Algorithm**

1. Train an LSTM-based regression model on the given dataset (Table 1).
  - The input features (*X*) are the scores prior to the final exam, i.e. quizzes, the midterm exam, project, and others.
  - The output (*y*) is the final exam score.
2. Calculate the error between the scores predicted by the trained model and the actual exam scores. Apply a KDE-based outlier detection method on the set of errors to determine the abnormal scores.

## 4 Numerical experiments

In this section, we conduct numerical experiments to test the performance of the proposed algorithm against other benchmark methods. The experiments are carried out over a range of scenarios. The data used in the experiments consists of four synthetic and one real-life datasets. We employ a naive as well as more sophisticated anomaly detection strategies as benchmarks against the proposed method. To measure the efficacy of the algorithms, we compute the true positive and false positive rates of the classification results. The results of the experiments show that the proposed method outperforms the benchmark methods in the majority of the scenarios. The proposed algorithm achieves an average of 95% true positive rate and 2.5% false positive rate on the synthetic data. It achieves 100% true positive rate and 4% false positive rate on the real-life data. The results of the experiments are a strong indication that the proposed algorithm may be an effective tool in fraud identification.

### 4.1 Benchmark methods

The naive strategy employed in practice is to compare the mean of the scores prior to the final exam with the score on the final exam. If the final exam score is significantly higher than the mean score of the preceding assessments, then it would be a sign of a potential violation. It is a simple and intuitive approach commonly used by instructors to identify potential cases of fraud. Despite its simplicity the naive strategy can be a quick and effective tool to detect abnormal scores on the final exam. As such, it is a good baseline strategy that we aim to beat. Further to the baseline method, we benchmarked our proposed algorithms to the following standalone outlier detection techniques: robust covariance [33], isolation forest [45], and local outlier factor [36]. The benchmark methods are implemented in the popular scikit-learn machine learning library. We used the default parameter settings for the models with exception of the contamination parameter which was set to 0.09. Note that in the sklearn implementation, the default value of the contamination parameter is set to 0.1, i.e., 10% of the points are deemed outliers.

It is important to note that in our data, a sequence of grades is labeled based on whether or not the student cheated on the final exam. This information is built into both our data generation and in our cheating detection method, but is wholly unavailable to the baseline methods. In other words, the baseline methods do not make the same assumption about the structure of the data that is available to the proposed method. Thus, the comparison is not entirely fair.

### 4.2 Datasets

**Fig 3:**
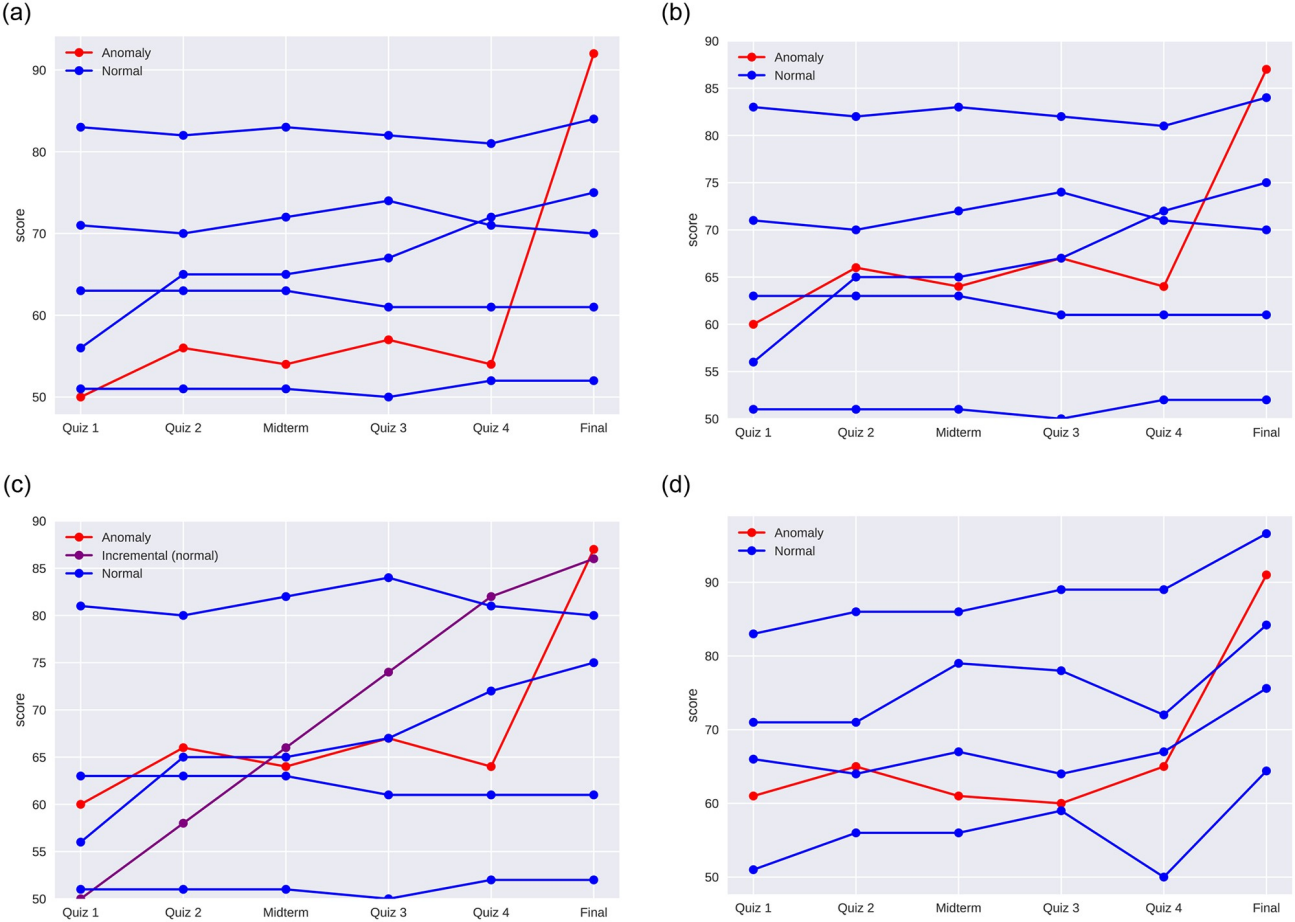
Representative samples of the simulated datasets used in our experiments. The datasets capture different scenarios for the distribution of the grades. (a) A representative sample of Dataset 1 grades. The dataset consists of 91% normal and 9% anomalous grades. The normal grades consist of three quarters homogeneous grades and one quarter increasing grades. The anomalous grades *rise sharply*—by 35 points—during the final exam. (b) A representative sample of Dataset 2 grades. The dataset is similar to Dataset 1. However, the anomalous grades rise *less sharply*—by 20 points—during the final exam. As a result, the outliers are harder to identify. (c) A representative sample of Dataset 3 grades. The dataset is similar to Dataset 2. However, around 10% of the normal grades are increasing at an incremental pace so that the difference between the average prior and final exam scores are same as in the anomalous instances. As a result, it is even more challenging to identify the outlier scores. (d) A representative sample of Dataset 4 grades. The dataset is designed to simulate a scenario when the final exam is easy and *everyone* receives a relatively high grade. The normal final exam scores 10 points higher than the average on prior assessments. The anomalous final exam scores 25 points higher than the average preceding scores.

The experimental evaluation of our proposed approach includes four different synthetic and one real-life datasets. The synthetic datasets are designed to simulate real-life scenarios of exam cheating. A representative sample of each synthetic dataset is shown in Fig 3. Dataset 1 consists of 100 normal grades and 10 cheating cases (Fig 3a). The cheating cases are designed to be egregious with a 35-point difference between the average of the regular assessment scores and the final exam score. The normal grades are mostly homogeneous. Approximately 80 of the normal grades remain consistently within a 10-point range across all assessments including the final exam. The remaining 20 normal grades increase during the semester. The cheating cases in Dataset 1 are relatively easy to identify even with the naive strategy of comparing the average grade prior the final exam to the score on the final exam. Dataset 2 is similar to Dataset 1. However, the cheating cases are more masked in Dataset 2 with only a 20-point difference between the average score prior to the final and the score on the final exam (Fig 3b). A smaller margin between the final exam and preceding scores would make it more difficult to discern the cases of cheating. As a result, the performance of the anomaly detection algorithms deteriorates. Dataset 3 is similar to Dataset 2. However, Dataset 3 contains normal grades that are rising throughout the semester in such a way that the difference between the average score prior to the final exam and the final exam is the same as in the cheating cases. As shown in Fig 3c, the scores in maroon color are steadily rising indicating a normal pattern. On the other hand, the scores in red color increase abruptly with much larger amount on the final exam. As mentioned, the difference between the average score prior to the final and the score on the final exam is similar in both instances. To distinguish between the former and the latter cases traditionally requires human judgment. Nevertheless, we show that the proposed method can automatically detect cheating cases even under such challenging conditions. The last dataset in our experiment illustrates the scenario of an easy final exam where all the grades increase relative to the regular assessments. The normal grades are simulated to increase by 10 points on the final exam over the average of the preceding scores. The cheating cases are designed to increase by 25 points on the final exam compared to the prior regular semester assessments (Fig 3d). Since all grades increase on the final exam it becomes more challenging to identify cases of cheating. Further details about the synthetic datasets together with the code can be found on our public GitHub repository (https://github.com/group-automorphism/exam_cheating).

In addition to the synthetic data, we also employ one real-life dataset in our experiments. Although we would have liked to use more real-life data it is difficult to obtain due to a number of reasons. Our dataset consists of 52 observations of which 3 are positive cases. Each observation includes scores from 4 quizzes, a midterm exam, and the final exam.

### 4.3 Results and discussion

To determine the efficacy of the algorithms, we calculate the true positive rate (TPR) and the false positive rate (FPR) of the classification results. The TPR measures the proportion of actual positives that are correctly identified as such. The FPR is the proportion of all negatives that are identified as positive by the test. For each synthetic dataset, the experiment is run 20 times. We report the mean and standard deviation of the TPRs. The results of the experiments reveal that the proposed algorithm may be an effective tool in identifying cases of cheating on the final exam based on prior scores.

The mean TPRs of the proposed algorithm together with the benchmark methods is presented in Table 2. Results indicate that the proposed algorithm has the highest overall average TPR of 0.874 among all the tested algorithms. In addition, the proposed method produces the highest individual mean TPR on Datasets 2 and 3. The standard deviations of the TPRs are also presented in Table 2. The standard deviation of the TPRs range between 0.05 and 0.12.

**Table 2:** The mean and standard deviation TPR for the anomaly detection algorithms. The results represent experiments on four datasets based on 20 simulated experiments. The proposed method (*NewAlgo*) produces the best overall results.

	DS1	DS2	DS3	DS4	Overall
Naive	1.000±0.00	0.780±0.112	0.490±0.158	1.000±0.000	0.818
RobustCov	0.975±0.043	0.420±0.194	0.020±0.068	0.450±0.470	0.466
IsoForest	0.24±0.132	0.000±0.000	0.000±0.000	0.015±0.036	0.065
LOF	0.040±0.058	0.045±0.074	0.055±0.086	0.055±0.092	0.049
NewAlgo	0.915±0.111	0.760±0.097	0.840±0.107	0.980±0.051	0.874

The FPR of the proposed algorithm together with the benchmark methods is presented in Table 3. As can be seen from the table, the proposed algorithm produces the smallest overall average FPR of 0.013 among all tested detection methods. It also produces the lowest FPR on Dataset 2 and Dataset 3.

**Table 3:** The mean and standard deviation of FPR for the anomaly detection algorithms. The results represent experiments on four datasets based on 20 simulated experiments.

	DS1	DS2	DS3	DS4	Overall
Naive	0.000±0.000	0.022±0.011	0.051±0.016	0.000±0.000	0.018
RobustCov	0.002±0.004	0.058±0.019	0.098±0.007	0.055±0.047	0.053
IsoForest	0.076±0.013	0.100±0.000	0.100±0.000	0.098±0.004	0.094
LOF	0.096±0.006	0.096±0.007	0.094±0.009	0.094±0.009	0.095
NewAlgo	0.008±0.011	0.024±0.010	0.016±0.011	0.002±0.005	0.013

To further test our proposed algorithm, we applied it to simulated data of size 220 which is double the original size used in the preceding experiments. We simulated each dataset 20 times and recorded the mean TPRs. The results of the experiments with the increased class size are largely in line with the original results. As shown in Table 4, the mean TPR values of the proposed method range between 0.780 and 0.988. The new algorithm achieves the highest overall mean TPR of 0.872 among all the tested methods. The new overall average TPR is close to the original overall average (0.868).

**Table 4:** The mean TPR for the anomaly detection algorithms. The results represent experiments on four datasets based on 20 simulated experiments and the class size of 220. The proposed method (*NewAlgo*) produces the best overall results.

	DS1	DS2	DS3	DS4	Overall
Naive	1.000	0.755	0.515	1.000	0.818
RobustCov	0.972	0.402	0.022	0.267	0.416
IsoForest	0.280	0.008	0.002	0.038	0.082
LOF	0.092	0.053	0.038	0.042	0.056
NewAlgo	0.915	0.780	0.807	0.988	0.872

The primary factor in identifying the cases of cheating is the score on the final exam relative to the prior assessments. In addition, secondary factors such as the trajectory (momentum) of the scores and the performance relative to the rest of the class must be considered. Although the naive approach does not take into account the trajectory and the relative performance, it does capture the average difference between the prior assessments and the final exam scores. As a result, it produces a robust performance. On the other hand, the standard outlier detection methods are based purely on the geometry of the sample points. The points away from the main cluster or the points in a relatively low-density region of the sample space are labeled as outliers. No special meaning is assigned to any particular feature.

To better understand the results of the numerical experiments (Tables 2–4) and to illustrate the mechanism of the outlier detection methods consider the dataset of grades consisting of Quiz 1, Quiz 3, and the Final exam scores from DS2 dataset (Fig 3b). The details of the cases of cheating and the outliers are provided in Table 5. As shown in Table 5, in the cases of cheating the scores on Quiz 1 and Quiz 2 are relatively stable before suddenly jumping on the Final exam. A sudden increase in the score is an indication of a potential case of cheating. The RobustCov method is a model-based method that fits a Gaussian ellipsoid to the dataset using the central data points. As shown in Table 5, the RobustCov method selects the points with a positive trend in scores. Although it could be argued from a geometric stance that the points with a positive trend could be outliers, it would not be appropriate to make such an assumption from an academic stance. A student who consistently improves his/her marks between assessments and ultimately receives a high grade on the final exam is not surprising. The RobustCov method fails because it is unable to take into account the context of the problem. The IsoForest method is based on the random forest classifier. It operates on the principle that anomalous points have shorter paths from the root to the terminal node. As can be seen from Table 5, the IsoForest method labels as outliers the points with either high or low-valued elements. It appears that the points with extremely low or extremely high-valued elements require the fewest number of splittings to be isolated. As a result, the IsoForest method misses the cheating cases. The LOF method is based on the idea of selecting the samples that have a substantially lower density than their neighbors. It selects the points that are away from clusters. The cheating cases are not identified because they form a cluster of points with low quiz scores and high final exam score. In addition, the cheating cases are close to the cases where students gradually improve their marks making them even harder to distinguish.

**Table 5:** The scores of the (true) cheating cases and the outlier cases determined by the detection methods in DS2 dataset.

Cheating	Quiz 1	66	60	64	69	62	63	65	68	66	68
	Quiz 3	67	69	62	68	69	64	67	64	66	68
	Final	89	86	84	88	87	87	81	80	88	83
RobCov	Quiz 1	50	51	50	50	50	50	51	52	50	63
	Quiz 3	68	65	64	61	70	71	71	70	68	64
	Final	79	77	78	77	79	76	79	79	78	87
IsoForest	Quiz 1	54	59	58	57	54	87	89	83	88	50
	Quiz 3	57	55	51	51	51	89	85	89	89	56
	Final	51	50	58	54	52	81	87	89	80	60
LOF	Quiz 1	69	78	79	82	87	89	88	53	59	53
	Quiz 3	69	77	78	89	89	85	89	67	71	66
	Final	65	79	75	80	81	87	80	70	76	68

The results of the experiment on the real-life data are in line with the results on simulated data. As shown in Table 6, the proposed method attains the highest TPR. Indeed, the proposed method successfully identifies all 3 cases of cheating among 52 cases. The proposed method also attains the lowest FPR. Only 4% of students who did not cheat were flagged as suspicious.

**Table 6:** The true positive and false positive rates of the anomaly detection algorithms. The results represent experiments on a single real-life dataset. The proposed method (*NewAlgo*) produces the best overall results.

	Naive	RobustCov	IsoForest	LOF	NewAlgo
TPR	0.67	0.33	0.33	0.67	1
FPR	0.06	0.08	0.08	0.06	0.04

The results of the numerical experiments reveal that the proposed algorithm achieves high levels of accuracy. It can identify almost all cases of cheating while avoiding falsely labeling a normal score. The proposed method significantly outperforms the benchmark methods both in TPR and FPR. The performance of our method is relatively consistent across different datasets.

In the problem of identifying the potential cases of cheating, the final exam scores play a crucial role. The proposed algorithm is tailored to compare the predicted and the actual final exam scores to identify the anomalous grades. The standard outlier detection algorithms have the added burden of learning the importance of the final exam scores from the data and therefore do not perform well.

## 5 Conclusion

The COVID-19 pandemic has forced most of the schools and universities in the world to switch to online education. In particular, exams have been administered remotely with little supervision. As a result, the likelihood of exam cheating has increased dramatically. The main objective of the present research was to examine a new approach to detect potential cases of cheating using machine learning techniques. In particular, we considered the problem of identifying cases of cheating on the final exam. We propose a novel method for identifying potential cases of cheating on the final exam using a post-exam analysis of the student grades. Our method takes into account student grades prior to the final exam, grades on the final exam, and the overall performance of the class to make a decision. We employ LSTMs in combination with a KDE-based outlier detection technique to identify the potential cases of cheating. The insights gained from this study may be of assistance to academics and administrators interested in preserving the academic integrity of course assessments.

The proposed method yields promising results on various datasets. It achieves an average TPR of 0.95 and FPR of 0.05 in our numerical experiments. Our method significantly outperforms the benchmark methods that were used in the experiments. However, it is important to note that the baseline algorithms have the added burden of learning the importance of the final exam scores from the data. So the comparison may not be entirely adequate. The performance of the proposed method is consistent across different scenarios simulated in our experiments and produces near perfect accuracy in identifying cases of cheating on the data used in the study.

The present study lays the groundwork for future educational research into outlier detection techniques, as it proposes a complement to commercial plagiarism detection software and possibly a non-intrusive deterrent alternative to highly-polemical remotely-invigilated exams. The reader should bear in mind however that the scope of this study was limited in terms of sample size and context, its replication at other universities would undoubtedly be valuable and recommended. Further investigation and experimentation into the proposed method and academic integrity detection is therefore strongly advocated. Remote exam administration poses a great challenge to preserving the academic integrity of the exam. It is relevant issue today and it will remain important in the future. Our method offers a great tool to help address the issue of academic integrity for remotely administered exams.

A natural progression of this work would be to observe the performance of our model by replacing the regression component with alternatives such as linear regression or Gaussian process regression. Indeed, it would be informative to test different regressor/anomaly detection combinations and perhaps even obtain an improvement over the current configuration.

## Supporting information

S1 Data (CSV)
